# Applicability of the model of inclusive education in early childhood education: a case study

**DOI:** 10.3389/fpsyg.2023.1120735

**Published:** 2023-07-21

**Authors:** Pille Nelis, Margus Pedaste, Carolina Šuman

**Affiliations:** Institute of Education, University of Tartu, Tartu, Estonia

**Keywords:** inclusive education, early childhood education, model, implementation, case study

## Abstract

Despite the development of policies and research supporting it, inclusion remains a challenge in contemporary education. We have developed a theoretical model for implementing inclusive education, thereby supporting early childhood education quality. It is necessary to establish the applicability of this model in order to apply it to improve the practices for adopting inclusive education. We conducted a case study, which showed that all levels and key characteristics of the theoretical model were also relevant in practice. However, as a result of the case study, the features describing the key characteristics were modified compared with the initial model. Additionally, the case study revealed that some of the features did not appear in practice. Those undetected features were mostly related to understanding the concept of inclusive education and the philosophy of inclusion. There appeared a need for a clearer understanding of inclusion on both the institutional and state level. The implementation of inclusive education does not in itself always increase inclusion or reduce exclusion. Therefore, when implementing inclusive education, it is necessary to think carefully about what is being done to allow all children to be meaningfully involved in the same classroom and by their teachers.

## 1. Introduction

The implementation of inclusive education remains a challenge in contemporary education systems despite having been a major area of interest in educational research during the last three decades (Ainscow, 2020; Haug, 2020; Lesar and Mihelič, 2020) and the basic idea in most international education policy documents (UNESCO, 2008; European Commission, 2014; European Commission/EACEA/Eurydice, 2019; Schleicher, 2019). There seems to be a gap between formulations and realizations of inclusive education (Haug, 2017). Therefore, we need a clear concept of inclusive education and an understanding of the factors that affect the implementation of inclusive education in order to provide quality early childhood education.

When creating favorable conditions for implementing inclusive education, one should consider both the holistic context and the key characteristics that occur in this context. The diversity of key characteristics across early childhood settings points to a need for research into the practical implementation of inclusive education in contextualized and multifaceted ways. Such research would provide a better understanding of how inclusive education could be differentially implemented across multiple early childhood contexts while maintaining the quality of its key features (Love and Horn, 2021). Several models and approaches have been used to systematically describe the key characteristics for implementing inclusive education (Odom et al., 2004; Fyssa et al., 2014; Pelatti et al., 2016; European Agency for Special Needs and Inclusive Education, 2017). However, these models have a number of shortcomings. For example, they are not sufficiently operationalized for implementation and seem to focus on characteristics implemented only at the classroom level. Also, none of the models define inclusive education (Nelis and Pedaste, 2020). A model that includes the concept of inclusive education and evidence-based practice on all levels of the education system could fill this gap in the field of inclusive education. Based on a systematic literature review, we propose a model that involves the general definition and key characteristics of inclusive education in early childhood education (Nelis and Pedaste, 2020). In this model, contemporary inclusive education is operationalized by 14 key characteristics on five levels. Next, we introduce both the definition and model used in the current study.

### 1.1. Concept of inclusive education

Since the Salamanca Statement (UNESCO, 1994), an international consensus has existed concerning the need to provide equal educational rights to all people with varied special education needs. The most frequent discussion points related to the concept or definition of inclusive education are its narrow and broad definitions (Haug, 2017). The narrow definition includes only children with special needs (Haug, 2017; Ainscow, 2020). This approach is concerned with placing children with special needs into mainstream settings, making them full members of regular groups. At the same time, it has been argued that inclusive education should not focus only on placement but also on meaningful participation and personalized support that would allow each child to realize their full potential (Love and Horn, 2021). The broad definition includes all marginalized groups (UNESCO, 1994; Haug, 2017), meaning that all children who might otherwise be excluded because of their diversity (special needs, gender, ethnicity, culture, social background, etc.) should be focused on and included in mainstream settings. Leijen et al. (2021) also indicate two narratives in contemporary inclusive education: “inclusion for some” and “inclusion for all.” They find that these two discourses can be bridged in the sense that one of them advocates for what ought to be long-term goals for the implementation of inclusive education, whereas the second verbalizes practical constraints and barriers that need to be overcome to make inclusive education real. In this study, we used the narrow concept of inclusive education. This concept is also adopted in the Estonian National Curriculum for Pre-School Child Care Institutions, where it is defined as education for children who need adjustments in their environment (playing and teaching aids, rooms, methods, etc.) or in the activity plan of the group due to their development needs, which arise from their abilities, state of health, linguistic or cultural background, or other personal characteristics.

In order to support inclusion and reduce exclusion, various aspects of the education context should be noted (Booth and Ainscow, 2002; Slee, 2005), for example, knowing the specifics of all marginalized groups. On the one hand, this makes the literature and practice in this field rich but, on the other hand, difficult. Legislation and the language we use to talk about inclusive education could lead to exclusion rather than inclusion. Some critics have argued that because people with disabilities are automatically included in universal human rights instruments, no separate initiatives are necessary and that specific campaigns may, in fact, have the opposite effect (Slee, 2005). Although the language of special educational needs can be a barrier to the development of inclusive practice, it remains part of the culture and policy framework and influences a variety of practices (Booth and Ainscow, 2002). For example, rather than using the term “special educational needs coordinator,” alternative terms such as “learning support coordinator,” “learning development coordinator,” or “inclusion coordinator” would be more appropriate. The use of such terms supports the inherent approach of inclusion of supporting all learners with difficulties rather than solely focusing on learners with special needs—which actually contributes to exclusion. Barriers also exist in students’ interactions and what and how they are taught. Barriers to learning and participation can prevent access to learning or limit participation within it (Booth and Ainscow, 2002).

Different authors (Slee, 2005; Ballard, 2013; Ainscow, 2020) have, over time, focused on the discourse on special needs as one of the obstacles to implementing inclusive education. As long as the discourse on inclusion is used to protect the professional interests of special needs education, full inclusion is at skate. As long as teachers keep to the concepts of mainstream and special needs students, implementation of the concept of inclusive education is not supported (Slee, 2005). Both Andrews et al. (2021) and Brown (2005) also suggest that labeling children with special needs leads to exclusion because it emphasizes the need for professionals and may be why teachers claim that they are not adequately prepared to teach those children. In addition, the need for professionals refers to the expectation that those children require individual activities carried out separately from other children. Individualization applied in this way results in excessively distinguishing and separating the special needs child. In this context, individualization becomes synonymous with exclusion. Ballard (2013) suggests that to reduce exclusion and create more inclusive ways of living, we need to think differently and consider alternative ideas that would support the development of fairer and more democratic practices. New ways of thinking must be justified and understood in terms of philosophy, evidence, and values. Otherwise, they will be readily assimilated into traditional structures, just as the field of special education now uses the language of inclusion to modify itself in an effort to maintain control of policy and practice (Ballard, 2013).

Overall, this brings us back to the conceptualization of inclusive education and indicates the need for a universal definition of inclusive education and its interpretation in a way that increases inclusion and reduces exclusion. According to Haug (2017), despite a formal normative consensus, finding one universal definition of inclusive education is impossible. Florian (2014) has pointed out that, although no definition of inclusion has been universally accepted, developing a universally accepted definition may represent a positive step toward developing an inclusive practice. Mitchell (2015) has interpreted inclusion as a concept with multiple underlying values and processes. They provide a more realistic interpretation that considers the school’s complexity and the multiple mutually influential values underlying the concept. We have provided a definition that includes both the philosophical and practical sense of inclusion and involves aspects that have appeared in different definitions in the theoretical literature, thus aiming for a rich and universal approach (Nelis and Pedaste, 2020). Based on an extensive systematic literature review, inclusive education can be defined in a philosophical and a practical sense. The philosophical sense has been described in terms of four aspects: access, belonging and membership, social integration, and human rights. The practical sense has been described in terms of three aspects: participation, support, and development of every child. As a result of this study, a new definition of inclusive education has been provided:

An educational approach that takes into account human rights and provides all children with access to high-quality education in a learning environment where children feel social integration and belongingness in their wider social network despite their diversity; it is achieved by meaningful participation of all children and personalized support in the development of each child’s full potential (Nelis and Pedaste, 2020, 162).

In interpreting this definition, we can first point out that inclusive education is an *approach* to education. Secondly, the subjects of inclusive education are children—or, in a wider context—learners who perceive themselves in a specific way, whereas realizing each child’s (learner’s) full potential is the goal. Thirdly, it refers to the methods—active, meaningful participation and personal support—by which the approach, perception, and potential are realized. The current study is based on this definition.

### 1.2. Contemporary model for the implementation of inclusive education

**Figure 1:**
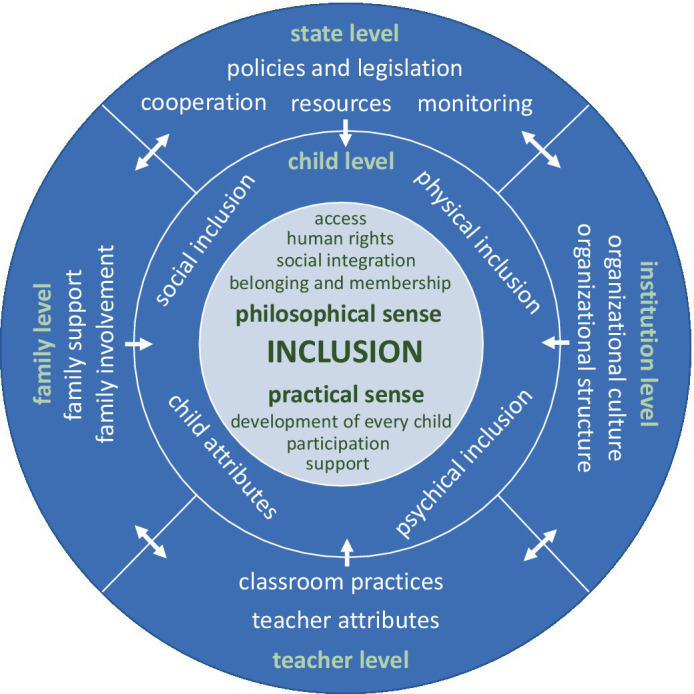
The model for the implementation of inclusive education in early childhood education (based on Nelis and Pedaste, 2020).

Another basis for the current study is the model describing different key characteristics of inclusive education. We have used a model (see Figure 1) that includes the general definition and key characteristics of inclusive education based on a systematic literature review (Nelis and Pedaste, 2020). In this model, contemporary inclusive education is operationalized by 14 key characteristics on five levels: *child level*, *teacher level*, *family level*, *institution level*, and *state level*. The first version of the model has been modified. The changes are explained below.

The model consists of three circles. The definition of inclusive education is at the center. This circle is divided into two parts showing the two dimensions of the definition: the philosophical and the practical sense (Lundqvist et al., 2015), accompanied by their specific features. Both dimensions of the definition should be understood and interpreted in the same way by all related stakeholders. It is highly recommended that a similar understanding of inclusive education be reached; this supports the implementation of inclusive education (Sukumaran et al., 2015; Florian et al., 2017). Effective and high-quality implementation starts from the approach and mindset toward inclusive education, requiring discussion, cooperation, and inclusion within and between different levels of the model.

Two other circles represent how the key characteristics of inclusive education in early childhood education are operationalized. At the center of each level is the subject related to the key characteristics or responsible for providing access and ensuring the quality of the key characteristics on a particular level, thereby influencing the implementation of inclusive education.

The arrows illustrate relatedness and influence between different levels of the model. On child level, the middle circle of the model, the outcomes are achieved through the implementation of inclusive education. All the other levels should consider the child’s attributes and provide physical, psychical, and social inclusion of each child (e.g., deaf children need one type of support, children with a lower level of cognitive abilities need another type of support, and handicapped children yet another type). Child level is directly influenced by family, teacher, institution and state levels. The key characteristics of teacher level influence the child, family, and institution levels. Teacher level is influenced by family, institution, and state levels. The key characteristics of family level are essential for implementing inclusive education in early childhood education, influencing child, teacher, and institution levels. Family level is influenced by state, teacher, and institution levels. The key characteristics of institution level influence child, teacher, and family levels and are in turn influenced by teacher, family, and state levels. The key characteristics of state level influence all other levels and are influenced by all other levels.

Compared to the first version of the model, we have changed the arrows on state level. In the first version, the arrows on state level were unidirectional. In the improved model, they are bidirectional because other levels on the outer circle provide input to state level, thereby influencing the key characteristics of state level. In addition, the texts in the middle circle were moved so that it would be clearer that the arrows from the outer circle apply to all elements in the middle circle.

In conclusion, this model demonstrates the responsibility of each stakeholder and could therefore support the implementation of inclusive education. As the model is theoretical and developed based on the literature, this study focuses on the applicability of this model and improving it using wider feedback collected from practitioners in empirical studies.

### 1.3. The purpose of the study and research questions

The theoretical overview describes and explains the concept of inclusive education and the key characteristics of inclusive education according to the model developed based on the literature. The formulation of a new, broader definition provides a shared understanding and interpretation of inclusive education. The key characteristics presented in the model help us understand what should be done to support the implementation of inclusive education to achieve increased inclusion and reduced exclusion. As the model has been developed based on a literature review, research into inclusive education needs to establish the applicability of the model in practice in order to foster the implementation of inclusion in early childhood education. Therefore, the purpose of this study is to investigate the model’s applicability by using a case study and to explore to what extent the case study results reflect increasing inclusion and reducing exclusion. Two research questions were formulated:

1. How are the practice and policy of inclusive education expressed in the Estonian kindergarten based on the theoretical model of inclusive education?

2. To what extent do the results of the case study illuminate the processes of inclusion and exclusion in the Estonian kindergarten?

## 2. Methods

For the purposes of this paper, we chose the case study format. In the case study, we explored the implementation of inclusive education through detailed, in-depth data analysis involving multiple data collection methods characteristic of a case study, such as questionnaires, observation, semi-structured focus group interviews, and document analysis (Miles and Huberman, 1994). The case study was carried out in the context of Estonia. In Estonia, early childhood education is characterized by a decentralized education system where kindergartens have great autonomy (Koolieelse lasteasutuse seadus, 1999), state-level legislation, and qualified kindergarten staff. The main principles of the schooling and education process are a high-quality child-centered learning approach (learning through play and child-initiated activities) and an individualized service model (Koolieelse lasteasutuse riiklik õppekava, 2008). Each kindergarten prepares and develops its own curriculum based on the national curriculum for pre-school childcare institutions considering the specific nature of the kindergarten. All children at least 18 months old are granted a place in an early childhood education and care institution (if the parents request it). Traditionally, there have been two teachers per group plus one assistant. Since 2015, it has been possible to have one qualified teacher, one assistant teacher, and one assistant per group.

### 2.1. Research approach

In our study, we provide a holistic description and better insight into the implementation of inclusive education. We conduct hermeneutic research, which strives to understand the wholeness of experiences within a context (Patterson and Williams, 2002). We opted for an exploratory case study to explore the phenomenon and provide depth to understanding it using a qualitative study design (Creswell, 2003). Additionally, hermeneutic research encourages researchers to outline their self-awareness of their role to understand how they interpret participants’ meanings and experiences; it also considers researchers to be primary instruments in the interpretative process (Patterson and Williams, 2002). The first author of this article has 17 years of experience in four kindergartens in several roles. The second author, an educational researcher, has 15 years of teaching experience from schools but has also served for several years as head of the board of trustees of the kindergarten (this is the body of the kindergarten management that includes a representative of teachers, representatives of parents of each group, and a representative of the rural municipality or city). The third author, a special educator, has 3 years of teaching experience in schools and 4 years in kindergartens: two as a support person in a special group and two as a support specialist in a kindergarten.

### 2.2. Participants

Purposeful sampling was used in this case study, whereby participants were chosen if they could purposefully inform an understanding of the research problem and central phenomenon in the study. One kindergarten was selected for the study based on the following criteria:

1. existence of support specialists in the kindergarten (at least a speech therapist or a special educator);
2. readiness to apply the principles of inclusive education in kindergarten; and
3. voluntary request to participate in the training course on inclusive education for kindergarten teams (the team had to include 1–2 representatives from the management, 1–2 support specialists, and up to five teachers).

These criteria were chosen because they support the implementation of inclusive education in kindergarten and are related to the purpose of this study. We selected one kindergarten that best met the selection criteria for the case study. The motivation of the kindergarten, as well as the fact that the implementation of inclusive education was their priority, was a decisive aspect of this selection. The selected kindergarten had already taken several steps to implement inclusive education: creating positions for additional staff, integration groups of children with special needs with other children and, introducing smaller groups. The participants from the kindergarten were selected by the kindergarten, and all participants were included in the study on a voluntary basis. To this end, kindergarten employees participating in the study and the parents of children participating were asked to sign an information and informed consent form. In the form, participants were informed of the purpose and all procedures of the study and that they were free to withdraw from the study at any time without consequence. The study was approved by the university’s ethics committee named “Research Ethics Committee of the University of Tartu” on 6th April 2021.

The selected kindergarten is a municipal kindergarten with a total of 165–175 children and 39 employees (18 pedagogical employees, including one principal, one head teacher, 15 teachers, and one speech therapist, as well as 18 employees who assist the teachers or manage the institution). The kindergarten has eight groups (see Table 1).

**Table 1:** Overview of groups in the kindergarten.

Type of the group	Number of groups	Number of children	Age of children (years)	Number of teachers	Number of teacher assistants
Creche group	1	14	Up to 3	2	1
Group for children between 3 and 5 years of age	2	22	3–5	2	1
		18		1	2
Group for children between 5 and 6 years of age	1	22	5–6	2	1
Group for children between 6 and 7 years of age	2	22	6–7	2	1
Integration group^*^	2	14	3–5	2	1
		18	6–7		

* The allowed maximum number in integration groups is smaller than in other pre-school institution groups with the consideration that one child with special needs fills three spaces.

The number of participants in the study differed due to the data collection method. A total of 14 completed questionnaires were received out of 18. Seven kindergarten employees [principal (P 1), director of studies (P 2), four kindergarten teachers (P 3, P 4, P 5, P 6), and a speech therapist (P 7)] participated in the focus group interview. The moderator was the first author of this article. The observation was carried out in one kindergarten group, including one teacher, one teacher assistant, and 17 children.

### 2.3. Data collection

To ensure the quality and trustworthiness of the study, we used triangulation of methods, where data are collected by several methods. This is natural in case studies in general, as it allows one to collect in-depth data on and conduct an in-depth study of the case (Miles and Huberman, 1994). We used a questionnaire, semi-structured focus group interviews, observation, and document analysis for data collection. The anonymity of all participants was granted, and we did not ask for any information that would reveal the participants’ identities.

#### 2.3.1. Questionnaire

The questionnaire identified aspects of the definition and key characteristics of inclusive education that are important for implementing inclusive education. The questionnaire was developed on the basis of the model of inclusive education in the context of early childhood education that was created based on the results of a systematic literature analysis (Nelis and Pedaste, 2020). It consisted of six sections. First, respondents were asked for background information (age, work experience, education, and knowledge of inclusive education). Second, respondents were asked to what extent were the key characteristics and features describing the key characteristics of (1) child level, (2) teacher level, (3) family level, (4) institution level, and (5) state level taken into account in the kindergarten. We used a five-point Likert-type scale, where all scale values were described as follows: 1 = to a very small extent; 2 = to a small extent; 3 = so-so; 4 = to a large extent; and 5 = to a very large extent. The online survey was then carried out via email invitations to the participants in April–May 2021.

#### 2.3.2. Focus group interview

The aim of the focus group interview in this study was to collect data on the key features and opportunities related to inclusive education that are used and/or followed in the implementation of inclusive education in kindergarten. The focus group interview was semi-structured. The interviewer used a script containing questions about the following subject blocks: organizational culture, organizational structure of the kindergarten, characteristics of teaching in an inclusive group, cooperation with parents, cooperation with non-kindergarten institutions, and policy and legislation. In addition, we focused on interpersonal relationships and participants’ culture of communication and behavior. The focus group interviews were carried out in May 2021. The interview was audio recorded. Before the interview, participants’ consent for recording was again requested. The length of the focus group interview was 111 min.

#### 2.3.3. Observation

The aim of the observation in this case study was to find which features of inclusive education occur in the kindergarten’s schooling and education process and physical environment. The observation was carried out in May 2021. The observation was recorded with two video cameras placed on a tripod to record the entire group and all activities. The length of the observation was 2 h. The video recordings were stored on the IRIS Connect (2022) platform, which allows secure storage and analysis of video files in a password-protected environment. During the observation period, the researcher observed activities during the schooling and education process and other activities, such as getting dressed for going outside. The observation protocol was based on the Nelis and Pedaste model of inclusive education (Nelis and Pedaste, 2020). The observation protocol included the key characteristics of the child level and teacher level of the model and related features of these key characteristics. All features in the protocol were described in such a way that it was possible to understand which activity was associated with the features during the observation. For example, the key characteristic of the child level “physical inclusion” meant that the space, furniture, tools, and teacher-child ratio ensure every child’s active and meaningful participation in the regular group regardless of special needs. This was described in the protocol with reference to the following features: (1) organization of the physical environment, including tools and materials that meet the needs of children, (2) teacher-child ratio, and (3) meaningful participation of all children in an inclusive learning environment (active participation and engagement of all children, including mental and linguistic inclusion). During the observation and later when reviewing the video recordings, the researcher (first author of this manuscript) noted the activities that met the description of the features in the protocol.

#### 2.3.4. Document analysis

We used document analysis because it allows a systematic procedure for reviewing or evaluating documents (Yin, 1994). Documents provide data on the context within which research participants operate, information and insights derived from documents can be valuable additions to a knowledge base, and documents provide a means of tracking change and development (Bowen, 2017). The document analysis involved two documents, the curriculum and development plan of the kindergarten, chosen to acquire an overview of the features of inclusive education contained in them compared with the model of inclusive education.

### 2.4. Data analysis

The study employed a within-case analysis, meaning that a detailed case description was produced. The results of the questionnaires were analyzed using a quantitative analysis method—descriptive statistics. We calculated the frequency of the respondents’ answers to each question. In analyzing the interview, observation, and documents, we used qualitative deductive content analysis. In this theory-driven analysis, there is a framework behind the deductive approach of analysis where the system of categories is established before coding the text. The categories were built based on the Nelis and Pedaste model of inclusive education (Nelis and Pedaste, 2020). Two strategies were used to increase the study’s trustworthiness. First, two researchers (the first and the third author) assessed the appearance of 46 features of the model based on the focus group interview transcription. Code 1 and code 0 were used for this purpose. Code 1 means that the feature appeared in the interview. For example, code 1 for the feature “philosophy of inclusion” indicates a shared understanding of inclusion that includes clear principles and forms of inclusion. Code 0 means that the feature did not appear in the interview. The Cohen’s Weighted Kappa for assessing inter-rater reliability was not acceptable (Kw = 0.443). Therefore, the two coders discussed all cases where they had different opinions until they reached a consensus. The second strategy adopted to increase trustworthiness was peer examination. Specifically, the first author discussed the results of the analysis with the third author as well as interpretations of the data gathered.

The data were collected anonymously, and the participants cannot be identified based on the responses. Therefore, the results are not associated with any person or kindergarten. The results are presented in a generalized form.

## 3. Results

The results related to both research questions are presented on the basis of the five levels of the model in this chapter. The findings are presented based on data gathered with the questionnaire, focus group interview, observation, and document analysis. Results show that all key characteristics on five levels of the theoretical model—child level, teacher level, family level, institution level, and state level (see Figure 1)—are also evident in the case study. Each level comprises key characteristics and features that describe the key characteristics in greater detail. Below, we describe the results of each level based on the data collection method used. To answer the second research question, we looked at each level for examples in the data that describe inclusion and those that could promote exclusion.

### 3.1. Results of child level

At child level, all key characteristics presented in the theoretical model occurred in the case study. All features occurred as well, but differed depending on the method used. Different methods allowed us to see different aspects of the features, while some did not reveal some features at all. While the questionnaire and the focus group interview allowed us to see all key characteristics, the observation and document analysis only partially revealed the key characteristic *physical inclusion*. This meant that not all features described in the theoretical model occurred in this case study in relation to physical inclusion (see Table 2).

**Table 2:** Results of the child level by method.

Key characteristics and features	Quest	FG int	Obs	Doc an
Child attributes	X	X	X	X
- Specific child attributes	x	x	x	x
- Characteristics of special needs (type and severity)	x	x	x	x
Physical inclusion	X	X	V	V
- Environment management	x	x	x	-
- Teacher-child ratios	x	x	-	-
- Children’s participation in inclusive classroom	x	x	x	x
Social inclusion	X	X	X	X
- Positive social integration	x	x	x	x
- Positive attitude toward children with special needs	x	x	x	x
Psychical inclusion	X	X	X	X
- Acceptance	x	x	x	x
- Membership	x	x	x	x
- Health and safety (well-being)	x	x	x	x

Quest, questionnaire; FG int, focus group interview; Obs, observation; Doc an, document analysis; x, mostly occurred; V, partially occurred; -, did not occur.

**Figure 2:**
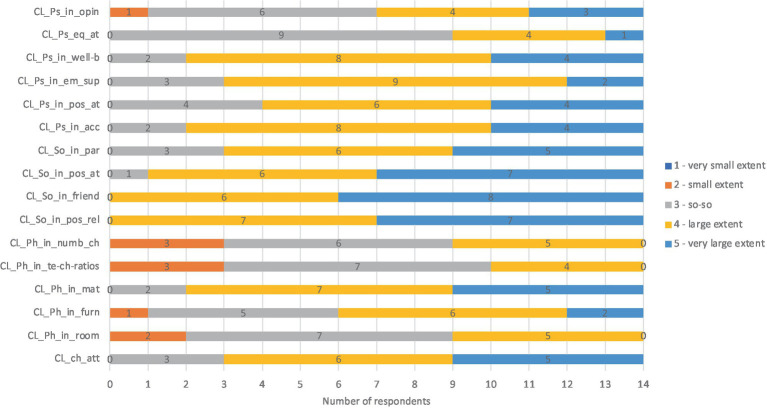
Results about key characteristics and features of child level. CL_ch_att, Child level, child attributes; CL_Ph_in_room, Child level, Physical inclusion, room; CL_Ph_in_furn, Child level, Physical inclusion, furniture; CL_Ph_in_mat, Child level, Physical inclusion, materials; CL_Ph_in_te-ch-ratios, Child level, Physical inclusion, teacher-child ratios; CL_Ph_in_numb_ch, Child level, Physical inclusion, number of children in group; CL_So_in_pos_rel, Child level, Social inclusion, positive relationships; CL_So_in_friend, Child level, Social inclusion, friendships; CL_So_in_pos_at, Child level, Social inclusion, positive attitude; CL_So_in_par, Child level, Social inclusion, participation; CL_Ps_in_acc, Child level, Psychical inclusion, acceptance; CL_Ps_in_pos_at, Child level, Psychical inclusion, positive attitude; CL_Ps_in_em_sup, Child level, Psychical inclusion, emotional support; CL_Ps_in_well-b, Child level, Psychical inclusion, well-being; CL_Ps_eq_at, Child level, Psychical inclusion, equal attitude; CL_Ps_in_opin, Child level, Psychical inclusion, opinions of a child.

The questionnaire showed that all four key characteristics of child level were considered in the implementation of inclusive education. The respondents rated the extent to which key characteristics and features of child level were considered in the kindergarten. According to most respondents, *child personal attributes* and features related to *social and psychical inclusion* were considered to a large extent (value 4) or to a very large extent (value 5). Some features of psychical inclusion – such as considering children’s opinions and giving them equal attention – were considered so-so (value 3). At the same time, the results showed that the features of *the child’s physical inclusion*, such as *the number of children in the group, teacher-child ratio*, and *the specifics of the room*, were considered to a smaller extent (values 3 and 2; see Figure 2).

As respondents could include their opinions in the questionnaire, some commented that a child with special needs, such as a child with a behavioral disorder or autistic traits, often required and received more attention than other children. In addition, according to respondents, dealing with a child with special needs requires more specific training from the teacher.

The focus group interview and observation showed that all key characteristics—*child attributes, physical inclusion, social inclusion*, and *psychical inclusion*—were important in the implementation of inclusive education by the participants of this case study.

Regarding *child attributes*, *a child’s personal attributes*, as well *as special needs*, were mentioned in the focus group interview. Participants mentioned a variety of special needs that they had encountered. There was a specific plan for child screening in case any problems were noticed. During the observation, it was apparent that some children acted faster, some needed more time, some were more modest and spent more time watching others, some were active and wanted to talk constantly, some needed more attention and guidance from the teacher, and so on. There was one child with special needs in the group who needed more attention from the teacher.

Regarding *physical inclusion*, participants mentioned in the focus group that *the number of children* and *teacher-child ratios* were important features in implementing inclusive education. Participants indicated that having a smaller number of children in the group supports the *meaningful participation of every child*, especially with special needs children. The participants themselves emphasized:

*“The more adults in the group, the better. Adult-child ratio. We no longer have twenty-four children in a group. That twenty-four is still too big a number to include all children, and in this sense, the first thing is still the number of children in the groups.”* (P 3)

The teacher can then consider each child’s developmental level and personal attributes and give children much more personal attention. Related to *physical inclusion*, an *appropriate environment* turned out to be crucial for inclusion. Observation made it possible to see that there were two different rooms, which allowed for activities in different groups. There were also places for a child to be alone and possibilities for individual work with children, working with smaller groups, and working with the whole group.

The focus group brought out that *social inclusion* means that all children are included in the games and activities during the whole day. However, special needs children were not always included in the games. One of the participants said:

*“I have the experience that other children do not really want to include a child with special needs in their games. They don’t push them out directly, but they find other ways to exclude this child.”* (P 5)

They The focus group also revealed that it was the teacher’s responsibility to find a solution and support friendship and positive interaction with peers in the group. Observation showed that *teachers’ attitudes* and behavior supported *social inclusion*. Friendly communication with and support for all children was evident during the observation. Also, adults in the group supported children’s friendships. For example, when dividing children into groups, the teacher considered the child’s wish to be in the same group as her friend.

Based on the focus group, getting to know the child was regarded by participants as important for *psychical inclusion*. Teachers considered the specifics of each child when designing and conducting the activities and made sure that all children could take an active part in the joint activities. The teachers treated all children equally and preferred no one. If necessary, a child with special needs was assisted. *Psychical inclusion* was also evident during the observation. All children were welcome in the group. Adults paid attention to each child and personally welcomed each child as they arrived in the room. They also checked that all children were welcomed into the games by other children. If necessary, they intervened and supported the child in finding a game. The teacher supported all children’s active participation in activities and teacher guided all children during the activities, if needed—including the child with special needs.

In document analysis, the key characteristics of child level were only partially detectable because some features were missing from the documents. Regarding *child attributes*, the kindergarten curriculum contained the principle that the personal attributes of all children would be considered. The concept of *a child with special needs* was also defined in the curriculum. According to the definition, “a child with special needs is a person whose developmental needs due to their abilities, state of health, linguistic and cultural background, and personality require changes or adjustments to the child’s growth environment or group action plan.” In addition, the documents also included the principles and organization of the schooling and education process for a gifted child. Regarding *physical inclusion*, only the feature *participation in an inclusive classroom* appeared in the documents. The general principles of the kindergarten’s curriculum supported the active and meaningful participation of every child. In the schooling and education process, the teacher’s responsibility was to consider the child’s individuality and development potential. At the same time, the features *environment management* and *teacher-child ratios* did not appear in the documents. The feature *social inclusion* appeared in the documents. The principles and organization of the schooling and education process and expected results of children’s development outlined in the kindergarten’s curriculum showed that *positive social integration* and *a positive attitude toward children with special needs* were important. For example, the curriculum called for increasing attention to be paid to group work skills in kindergarten and children learning to work and play together with their peers. The key characteristic *psychical inclusion* and features related to this key characteristic appeared in the documents. Tolerance, care, inclusion, and cooperation were presented as the main values of the kindergarten. Considering the specific attributes of each person was stated as one of its main principles – referring to the endeavor to make all children feel accepted and full members of the community. In addition, the kindergarten had joined the “Bullying Free Kindergarten” program and the Network of Health Promoting Kindergartens, which help to ensure children’s well-being in kindergarten.

#### 3.1.1. Inclusion and exclusion on the child level

On the child level, there were good examples of inclusion (e.g., teachers consider each child’s attributes, fewer children in the group, supporting friendship, and positive interaction with peers in the group), but the results also showed possible exclusion. For example, regarding social inclusion, the data showed that children with special needs were sometimes excluded from other children’s games. According to teachers, this type of exclusion occurred less in the group with fewer children. At the same time, respondents pointed out that how children are involved in the activities tends to depend on the teacher’s attitude and professionalism. There were some good examples of inclusion, specifically psychical inclusion. Respondents mentioned that the teacher paid equal attention to all children. According to them, inclusion was also supported in the kindergarten by considering the specific attributes of all children, as long as the child with special needs was not given too much special attention.

### 3.2. Results of teacher level

At teacher level, both key characteristics presented in the theoretical model appeared in the case study. All features appeared, but different methods allowed us to find different features on different generalization levels. In addition, one new feature, *professional development*, was added to the key characteristic *teacher attributes* (see Table 3) based on the case study results.

**Table 3:** Results of the teacher level by method.

Key characteristics and features	Quest	FG int	Obs	Doc an
Teacher attributes	X	X	V	X
- Personal attributes	x	x	x	x
- Competence (values, attitudes, knowledge and skills)	x	x	x	x
- Professional development	-	x	-	x
Classroom practices	X	X	X	X
- Quality of the learning process	x	x	x	x
- Setting of learning outcomes and goals	x	x	-	x
- Early identification of children with special needs	x	x	-	x
- Positive classroom climate	x	x	x	x
- Personalized activities	x	x	x	x
- Assessment of child development	x	x	-	x
- Reflection of teaching	x	x	-	x

Quest, questionnaire; FG int, focus group interview; Obs, observation; Doc an, document analysis; x, mostly occurred; V, partially occurred; -, did not occur.

**Figure 3:**
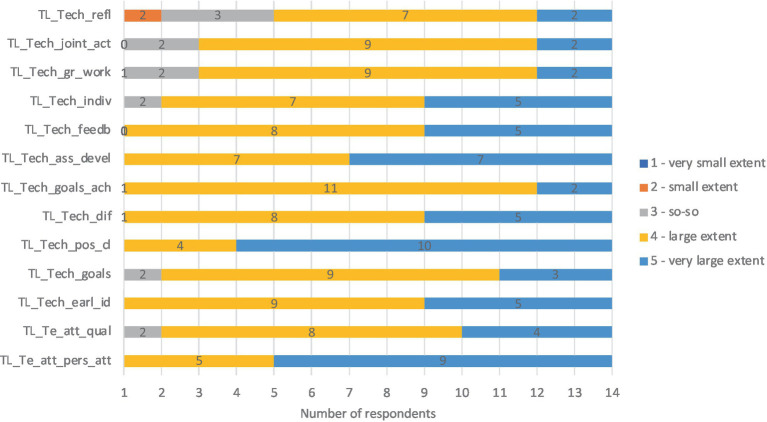
Results about key characteristics and features of teacher level. TL_Te_att_pers_att, Teacher level, teacher attributes, personal attributes; TL_Te_att_qual, Teacher level, teacher attributes, qualification; TL_Tech_earl_id, Teacher level, teaching, early identification; TL_Tech_goals, Teacher level, teaching, goals differentiation; TL_Tech_pos_cl, Teacher level, teaching, positive climate; TL_Tech_dif, Teacher level, teaching, differentiation; TL_Tech_goals_ach, Teacher level, teaching, goals achievement assessment; TL_Tech_ass_devel, Teacher level, teaching, assessing development of a child; TL_Tech_feedb, Teacher level, teaching, feedback; TL_Tech_indiv, Teacher level, teaching, individual tasks; TL_Tech_gr_work, Teacher level, teaching, group work; TL_Tech_joint_act, Teacher level, teaching, joint activities; TL_Tech_refl, Teacher level, teaching, reflection.

The questionnaire results indicate the relevance of all key characteristics and features of teacher level. According to the respondents, *the personal attributes* and *qualifications of the teacher* are essential in implementing inclusive education. Teachers carry out a variety of inclusive education activities to a very large extent (value 5) or to a large extent (value 4; see Figure 3).

The respondents added that the teacher’s values, skills, and commitment affect the quality and management of inclusion. Calmness, positivity, and concreteness were reported as the essential attributes of an early years teacher.

The focus group, observation, and document analysis showed that all key characteristics and features of the teacher level were relevant to implementing inclusive education. Both features of the key characteristic *teacher attributes—personal attributes and competence (values, attitudes, knowledge, and skills)*—were named by the participants. Flexibility, adaptability, responsibility, and a degree of self-criticism were considered important personal attributes of a teacher by the participants in the focus group. They said:

*“Today’s teacher must be very flexible and react quickly. You assess the situation, you notice, and then you have to be able to rethink something several times a day.”* (P 4)

*Teacher competence* is crucial in implementing inclusive education. Participants pointed out that teamwork and cooperation skills, as well as specific knowledge and skills related to special needs, are important for coping in an inclusive learning environment. Participants emphasized *teachers’ professional development*, for which several possibilities were mentioned: in-service training courses, literature for independent reading, online groups for discussions, sharing experiences and ideas for dealing with complicated situations and recommendations for literature, co-visions, learning in learning communities, and developmental discussions.

*Teacher’s personal attributes* and *teacher’s competence* were manifest in the teacher’s communication and behavior during the observation. The teacher communicated with the children in a friendly and supportive manner. She listened to them and answered their questions, explained the task clearly, and repeated the instructions if needed. She seemed positive and cheerful and stayed calm when one child refused to participate in the joint activities and started crying. She sat on the floor next to the child and talked to him until he calmed down and agreed to join the activities. The goals and activities were designed and conducted in accordance with the children’s age and developmental level.

The features *teacher’s personal attributes, competence*, and *professional development* were present in the documents. The kindergarten’s development plan contained a general principle that the employees have required relevant qualifications and that their attitudes correspond to the values of the institution. At the time, six teachers had senior teacher qualifications (according to the teachers’ professional standard). The kindergarten supported the professional development of its staff: attendance in higher education programs and in-service training courses was provided, and the amount of team training was increased. In addition, the kindergarten planned to develop the staff’s cooperation skills through various forms of teamwork and the introduction of best practices: teacher-to-teacher training, observation of teaching activities, and holding development discussions.

Features that describe the key characteristic *classroom practices* were mentioned during the interview. Although *the quality of the learning process* is a separate feature in the theoretical model, it was not specifically mentioned in the focus group interview. However, all the activities and features that the participants talked about referred to the quality of the teaching process: identifying special needs early on and creating an individual development plan for those children, setting individual learning goals to meet the needs of all children working in smaller groups and working individually with a child if needed, differentiating and adapting learning materials and activities, and giving additional time to children who need it. The participants explained:

*“Some children need more time, and even if you differentiate the tasks, they may need twice as long as other children. Well, we have also made it so that if others have a task, a child with a special need is a part of that task so that he can do it calmly.”* (P 3)

Assessment of children’s development is viewed as important. The teachers carry out observations and regularly take notes about children, and they reflect on their work individually or with another teacher. At the end of the school year, they write an analysis of the schooling and education process.

Observation revealed that *the classroom practices* involved features directly related to the teaching process: *positive classroom climate* and *personalized activities*. The teacher considered children’s specific attributes and planned manageable activities for all children. She gave tasks at different difficulty levels and guided the children if needed. The teacher engaged in a variety of activities: singing, discussing, listening, and practical activities. A positive classroom climate was created where children asked a lot of questions, worked together, helped each other, and gave positive feedback to each other. *Assessment of children’s development* and *reflection on teaching* were not visible during the observation process.

*Classroom practices* appeared in the documents. The development plan and curriculum outlined the principles and organization of the schooling and education process, including *setting of learning outcomes and goals, positive classroom climate*, and *personalized activities*. For *early identification of special needs*, a cooperation network had been set up, and, if necessary, teachers, in cooperation with support specialists and parents, would draw up an individual development plan. When planning and conducting the teaching and education process, the child’s level of development and specific attributes were taken into account by teachers. *Assessing the child’s development* was part of the daily schooling and education process. The latter involved various types of activities and methods, allowing the child to be an active participant. The child could learn through imitation, observation, research, experimentation, communication, play, practice, and so on. *Teachers’ reflection on teaching* was part of classroom practice, and by the end of the academic year, teachers would prepare an analysis of the schooling and education process. *The quality of teaching* was assessed through filming, observations, and the analysis of open activities.

#### 3.2.1. Inclusion and exclusion on the teacher level

At teacher level, the principle of considering the specific attributes of each child in the planning and implementation of the schooling and education process can be seen as a positive example of inclusion. Inclusion occurs when the teacher follows this principle and refrains from applying any extra activities that distinguish one child from another. Distinguishing between children through individual work, additional support, or individual activities can promote exclusion. In addition, the results revealed that teacher’s competence and professional development were interpreted only as teacher’s knowledge and skills related to special needs children—highlighting the needs of one specific group of learners, which does not support inclusion.

### 3.3. Results of family level

At family level, both key characteristics presented in the theoretical model—*family involvement* with features and *family support—*were evident in the case study. Unlike other methods, observation did not allow us to see the key characteristics of the family level (see Table 4). As for the features, all of these were present, but different methods revealed different aspects of the features or showed the features to differing degrees of comprehensiveness.

**Table 4:** Results of the family level by method.

Key characteristics and features	Quest	FG int	Obs	Doc an
Family involvement	X	V	-	V
- Family-professional partnerships	x	x	-	x
- Family needs, family expectations	x	x	-	x
- Family perceptions of inclusion	x	-	-	-
Family support	X	X	-	X
- Parent education	x	x	-	x
- Individualized family service plan	x	x	-	x

Quest, questionnaire; FG int, focus group interview; Obs, observation; Doc an, document analysis; x, mostly occurred; V, partially occurred; -, did not occur.

**Figure 4:**
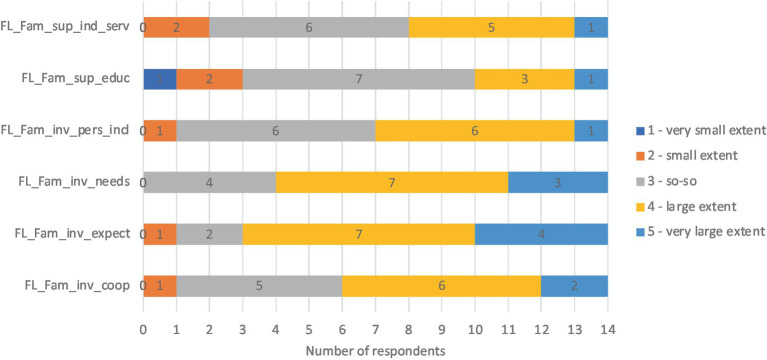
Results about key characteristics and features of family level. FL_Fam_inv_coop, Family level, family involvement, cooperation; FL_Fam_inv_expect, Family level, family involvement, family expectations; FL_Fam_inv_needs, Family level, family involvement, family needs; FL_Fam_inv_pers_incl, Family level, family involvement, family perceptions of inclusion; FL_Fam_sup_educ, Family level, family support, parent education; FL_Fam_sup_ind_serv, Family level, family support, individualized family service plan.

According to the questionnaire, both key characteristics were important for the implementation of inclusive education. Fourteen respondents answered the question about *family involvement and support*. The results showed that activities intended to involve parents were mostly implemented to a large extent (value 4) or to a very large extent (value 5). An interesting result was that five people gave vague (so-so, value 3) answers about cooperation. At the same time, the expectations and needs of parents were considered to a large extent (value 4) or to a very large extent (value 5). In terms of parental support, the results were average (value 3). Some respondents felt that the kindergarten applied activities for family support to a small extent (value 2; see Figure 4).

Respondents added that discussions with parents and development discussions enabled them to support the families.

The focus group interview and document analysis allowed us to detect both key characteristics of family level: *family involvement and family support*. In the focus group, family involvement included *family-professional partnerships, family needs*, and *expectations*. The partnership between the family and professionals started with active communication in different forms in the kindergarten: daily face-to-face communication and giving feedback about the child, multiple meetings during the academic year, individual discussions whenever difficulties occurred, development discussions, meetings between parents and support professionals, involving parents in the schooling and educational activities, and using parents’ knowledge of special needs to support the child with a specific special need. During the individual discussions, teachers discovered family needs and expectations for the kindergarten. Sometimes, cooperation was hampered by the fact that the parent would not see or recognize the problem.

Document analysis revealed that *family involvement*, such as *family-professional partnerships* and *family needs and expectations*, were valued in the kindergarten. The curriculum outlined the principles and organization of cooperating with parents, with several planned activities: daily discussions with parents, info boards, sharing information using email, kindergarten’s website, electronic schooling and education information system ELIIS, open days, and meetings and development discussions. Parents were also represented on the board of trustees. Although present in the theoretical model, the feature *family’s perception of inclusive education* was not mentioned in the focus group interview or revealed in the documents.

In the focus group, the participants pointed out that *family support* is important because parents often lack the knowledge and skills to manage their child’s special needs. On the other hand, they also added that sometimes the family is, in fact, well-prepared and aware of the matter. According to them, this is often the case when the child has been diagnosed at a very early age or before starting kindergarten. Training courses, literature, and recommendations for relevant groups outside of the kindergarten were offered by the kindergarten *for parent education*, as well as *an individualized family service plan* consisting of the services of a special educator and/or speech specialist, counseling by support specialists, and games and materials designed to support the child’s development.

In the documents, *family support* involved two features: *parent education* and *individualized family service plan*. These included the services of the speech therapist and, if necessary, an individual development plan for the child. There were different possibilities for parent education. Counseling in the schooling and education process took place through meetings with parents, daily conversations, and development discussions about the child. In order to raise parents’ awareness, workshops, training courses, round tables, events, etc., were organized in cooperation with the local government.

#### 3.3.1. Inclusion and exclusion on the family level

On the family level, there were many examples of increasing inclusion and reducing exclusion, for example, working with parents to identify the needs of each child and support each child’s development to help them reach their full potential. Development interviews were conducted with all families and their expectations were clarified. Several possibilities were used when cooperating with families: daily face-to-face communication and giving feedback about the child, multiple meetings during the academic year, individual discussions when difficulties occurred, development discussions, etc. The results revealed that exclusion may occur if cooperation is hampered by the fact that the parent does not see the problem with the child or does not recognize it. Here, participants considered it important that the teacher behaves and talks to the parents in the right way to prevent them from feeling excluded. The feature *family’s perception of inclusive education* did not appear in this case study, which could well be one of the factors contributing to exclusion because of unrealistic expectations from both family and kindergarten.

### 3.4. Results of institution level

At institution level, both key characteristics presented in the theoretical model—*organizational culture* and *organizational structure*—were present in this case study. As opposed to other methods, observation allowed us to only partially see the key characteristics of the institution level. With respect to the features, one feature related to *organizational culture*—*philosophy of inclusion*—was not evident in this case study. In addition, contrary to the theoretical model, the focus group interview indicated that—where staff-related activities were presented in a generalized way—more detailed features related to staff should be presented (see Table 5).

**Table 5:** Results of the institution level by method.

Key characteristics and features	Quest	FG int	Obs	Doc an
Organizational culture	X	V	V	V
- Sociocultural values and beliefs	x	x	x	x
- Philosophy of inclusion	x	-	-	-
- Various support systems	x	x	-	x
Organizational structure	X	X	-	X
- Leadership and administrative issues (policy documents and curriculum)	x	x	-	x
- Structure of employees (positions, number of employees, additional workforce)	x	x	-	x
- Work organization (work schedules, workload, work tasks)	x	x	v	x
- Benefits (benefits, bonuses, rest time and holidays)	x	x	-	-
- Different forms of inclusion	x	x	-	x

Quest, questionnaire; FG int, focus group interview; Obs, observation; Doc an, document analysis; x, mostly occurred; V, partially occurred; -, did not occur.

**Figure 5:**
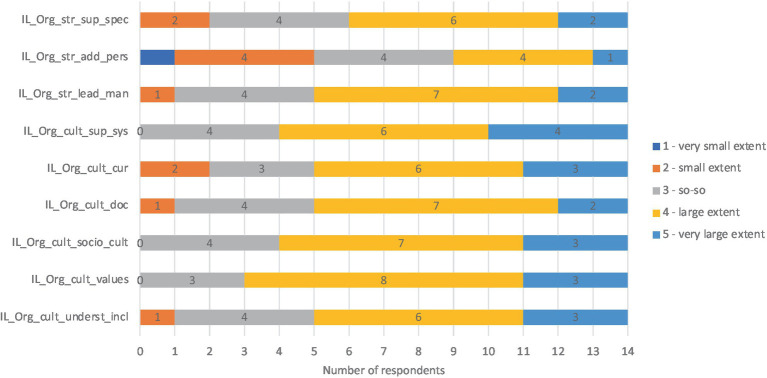
Results about key characteristics and features of institution level. IL_Org_cult_underst_incl, Institution level, Organization culture, creation of shared understanding of inclusion; IL_Org_cult_values, Institution level, Organization culture, shared values; IL_Org_cult_socio_cult, Institution level, Organization culture, socio-cultural approach; IL_Org_cult_doc, Institution level, Organization culture, inclusion in the documents; IL_Org_cult_cur, Institution level, Organization culture, inclusion in the curriculum; IL_Org_cult_sup_sys, Institution level, Organization culture, support system; IL_Org_str_lead_man, Institution level, Organizational structure, leadership and management; IL_Org_str_add_pers, Institution level, Organizational structure, additional personnel; IL_Org_str_sup_spec, Institution level, Organizational structure, support specialists.

The questionnaire revealed that both key characteristics clearly appeared in the kindergarten. Also, all features related to the key characteristics were considered. According to most respondents, the institution-level features of inclusive education were considered to a large extent (value 4) or, according to some, to a very large extent (value 5). However, most respondents felt that there were additional *staff-related issues* regarding the key characteristic *organizational structure*. According to most respondents, these features were considered to a small extent (value 4) or, according to some, to a very small extent (value 5; see Figure 5).

According to one of the respondents, the use of additional staff was related to resources. They also reported that the kindergarten had had five assistant teachers and one support person in past years but had to cut positions the previous year. At the time of the questionnaire, there were two assistant teachers and one support person. The kindergarten had reportedly created positions for support specialists but could not always fill them.

The focus group interview and document analysis revealed the relevance of both key characteristics of institution level in the kindergarten. During the observation, both key characteristics were only partially visible. In the group we observed, observation allowed us to see only the features *sociocultural values and beliefs* and work organization related to *staff-related issues*. Teamwork and *cooperation* between adults in the group seemed very good. The teacher and the teacher assistant did not share any information during the activity as they had previously discussed the whole activity and their roles in it. The collaboration worked well, and both knew what they had to do. The requirements for children were similar (both adults reminded them of the group agreements, e.g., putting things back where they belong).

In the focus group, the respondents found its *organizational culture* to be rather supportive of the implementation of inclusive education*. Sociocultural values and beliefs* were crucial and, according to respondents, shared responsibility among all kindergarten staff, a shared vision with explicit goals toward inclusive education, and working collaboratively were also important in the kindergarten. A supportive attitude toward inclusion was expected from all staff members and *various types of support* were implemented. The principles of a learning organization were also important in the kindergarten. There were peer-to-peer training sessions and teacher-led workgroups:

*“Each teacher has his or her own so-called trust teacher with whom concerns and problems are shared.”* (P 1)

There was cooperation between the groups. For example, there was a system of each group “being friends” with another group, with whom they would interact more and have various events together. New employees were assigned a mentor to support their integration into the organization. However, the focus group interview did not provide any information about *the philosophy of inclusion*: the participants did not discuss what inclusion meant for them or what they had in mind when talking about inclusion. They only talked about the inclusion of children with special needs.

The document analysis showed that the kindergarten’s *organizational culture* included *socio-cultural values* like kindness, tolerance, inclusion, and cooperation. The kindergarten’s development plan stated the aim to have a pleasant culture with shared values and orientation toward cooperation. Whereas *socio-cultural values* were described in the documents, *philosophy of inclusion* was still missing. *Various activities for support and cooperation* were mentioned in the documents. Supporting the development of a special needs child—including a gifted child—was seen as a team effort. The support team of a special needs child included teachers of the group, support specialists (physical and music teachers and speech therapists), a head teacher, and a parent.

According to respondents in the focus group, features related to *organizational structure*, such as *leadership and administrative issues*, were relevant to the kindergarten’s practices. Dealing with teachers’ workloads, extra staff, salaries, and benefits contributed to the successful implementation of inclusive education. While staff-related activities were generalized in the theoretical model, data from the focus group interview revealed that more detailed features could be presented separately here: *structure of employees (positions, number of employees, additional workforce); work organization (work schedules, workload, work tasks);* and *benefits (benefits, bonuses, rest time and holidays)*. The structure of the employees received a lot of attention in the kindergarten. Some groups had two teachers and one non-pedagogical employee. Other groups had one teacher, one teacher assistant, and one non-pedagogical employee. If necessary, the kindergarten could use additional workforce in order to support children with special needs. A lot of attention was paid to the organization of work. For example, non-pedagogical staff members were more involved in the schooling and education process in the group rather than cleaning or other tasks in the room. Teachers had flexibility in preparing their work schedules, giving them more autonomy and responsibility. As to the *various forms of inclusion*, most groups were so-called ordinary groups in the kindergarten, which include both normally developed children and those with special needs. There were also so-called integration groups. These also include both normally developed and special needs children but have a reduced number of children based on the principle that one child with special needs fills three places.

Based on the document analysis, *organizational structure* was apparent in the documents. There was *inclusive leadership and management* in the kindergarten. Various working groups had been set up to support development and teaching activities, and staff members were actively involved in development activities. Transmission of information and feedback was fast, with modern information technology possibilities used for this purpose. *Structure of the employees* was described in the documents as well as *work organization* and *benefits*. The kindergarten wished to create the positions of special education teacher and psychologist. The duties of each employee were specified in the job description. The kindergarten had developed its own recognition system. Regarding *various forms of inclusion*, description of different types of groups were outlined in the documents (see also Table 1).

#### 3.4.1. Inclusion and exclusion on the institution level

At the institution level, there were examples of both inclusion and exclusion. Values like tolerance and inclusion related to organizational culture that favored cooperation were supportive of inclusion. Cooperation and teamwork were common principles in the kindergarten. This kind of socio-cultural approach increases inclusion. The kindergarten’s curriculum also contained principles supporting the inclusion of all children, such as considering children’s specific attributes when planning and carrying out the schooling and education process. To increase inclusion, the principles of the kindergarten’s curriculum are discussed and shared by all teachers and have been meaningfully incorporated into the curriculum. The organization of teachers’ work also contributed to inclusion, with overlapping working hours encouraged—meaning that two teachers and a teacher assistant were working in the group simultaneously, thus supporting all children based on their specific attributes. The agreement to form groups of up to 20 children, four children less than the maximum number allowed by the state, also supported inclusion in the kindergarten. In addition, integration groups, which are even smaller, had been formed to increase inclusion in the kindergarten. At the same time, the main concerns regarding organizational structure, such as additional positions, were related to extra staff for children with special needs, indicating exclusion because of emphasizing one marginal group. If a support specialist supported all children, inclusion would be increased. The work organization of a speech therapist also referred to exclusion because a speech therapist deals with a child mainly outside the group, separately from other children.

### 3.5. Results of state level

At the state level, the theoretical model involves four key characteristics: *policy and legislation; cooperation; resources;* and *monitoring and evaluation*. The observation did not reveal any key characteristics of the state level, whereas document analysis revealed two key characteristics: *policy and legislation*—which was partially visible, as the feature *state/local context* was implicit in the documents—and *cooperation*—which was visible (see Table 6).

**Table 6:** Results of the state level by method.

Key characteristics and features	Quest	FG int	Obs	Doc an
Policies and legislation	X	V	-	V
- State/local context	x	x	-	x
- Local policy and law of inclusion	x	x	-	-
- Philosophy of inclusion	x	-	-	-
Cooperation	X	X	-	X
- Interagency support	x	x	-	x
- Collaboration between institutions	x	x	-	x
Resources	X	X	-	-
- Financial support	x	x	-	-
- Resources	x	x	-	-
Monitoring and evaluation	X	V	-	-
- Assessment of the quality of inclusion	x	x	-	-
- Stakeholders’ opinions	x	-	-	-

Quest, questionnaire; FG int, focus group interview; Obs, observation; Doc an, document analysis; x, mostly occurred; V, partially occurred; -, not occurred.

**Figure 6:**
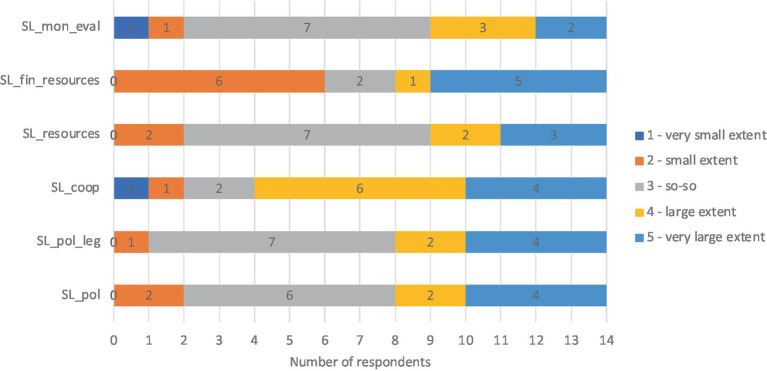
Results about key characteristics and features of state level. SL_pol, State level, policy; SL_pol_leg, State level, legislation; SL_coop, State level, cooperation; SL_resources, State level, mater; SL_fin_resources, State level, financial resources; SL_mon_eval, State level, monitoring and evaluation.

Based on the questionnaire, all the key characteristics of state level were crucial for implementing inclusive education. Regarding state-level key characteristics, we asked to what extent they supported the implementation of inclusive education in kindergarten. Most respondents rated as so-so (value 3) the extent to which the features related to key characteristics at state level supported the implementation of inclusive education in kindergarten. Some felt that state-level features supported implementing inclusive education to a very large extent (value 5) or to a large extent (value 4). The majority felt that working with external partners supported inclusion. At the same time, most reported that financial resources supported inclusion to a small extent (value 2; see Figure 6).

One respondent added that state level key characteristics are certainly very important and that, if inclusive education is already valued at state level and opportunities are created in the form of resources, it will be much easier for kindergartens to implement these opportunities.

The focus group interview revealed all key characteristics of state level. In the case *policy and legislation*, *the local context* was not directly mentioned in the focus group interview. It was implicit, however, as participants talked about unclear policies and parents’ low awareness of inclusive education and its positive effects. Regarding *the local policy and laws of inclusion*, it appeared that the principles of inclusive education were not sufficiently clear and unambiguously documented or were too rigid to implement inclusion. This, for example, restrains the flexible formation of groups while considering the specific attributes of the children and regulating the number of children in the group. *Philosophy of inclusion* as a separate feature did not appear in the interview. *Cooperation* includes *interagency support* and *collaboration between institutions* at the state level. Counseling centers at the local government level and Rajaleidja (the support center established by the state) were mentioned as support systems. Cooperation between all parties was seen as important but also a great challenge. Cooperation between the stakeholders in the support system was perceived as not efficient enough, and the whole process of supporting the child’s development in cooperation with different parties was seen as cumbersome and time-consuming. The interview revealed, first, that it is difficult for the parent who must visit various institutions and specialists, and second, that the right information does not reach the kindergarten. The respondents pointed out:

*“That the parent goes through different institutions many times and fills out different papers. That it would be nice if a parent could get all the necessary help in one place. That we have the parents run around different places, and in the end the parent can’t get help and we can’t get feedback.”* (P 7)

Respondents perceived a need to make the overall system more efficient for both families and kindergarten. According to respondents, *resources* included *financial support* and *other resources* on the state level. Financial support was mainly related to creating support specialist positions, e.g., psychologist, speech therapist, special education teacher, and support person. The low salary of support specialists was considered a bottleneck. Resources also meant better opportunities for supporting the professional development of kindergarten teachers, in the form of co-visions, for example*. Monitoring and evaluation* was viewed by respondents as a very important key characteristic at state level for ensuring the quality of inclusive education. They also found it a significant challenge because national level systematic data are collected only on the needs of support services in the kindergarten and on filling the positions of speech therapist and special education teacher. The state collects no other data, including information about access to special services in the kindergarten—one of the most critical indicators. No other indicators allow *assessing the quality* of and overall access to support services and inclusion in society. *Stakeholders’ opinions* are also missing in the process of state-level monitoring and evaluation.

Document analysis allowed us to find only two state-level key characteristics. First, regarding *policy and legislation*, *the local/state context* was apparent in the kindergarten curriculum. The curriculum outlined the principles and organization of the kindergarten’s schooling and education process based on the national curriculum and national pre-school institutions act: e.g., organization of schooling, concept of learning, and principles of schooling and education. The development plan gave an overview of the number of groups, types of groups, and number of children in the groups according to local law. Second, regarding *cooperation*—also discussed in the documents—the support system outside of the kindergarten included counseling centers at the local government level and state level. Two kindergarten integration groups had been created in cooperation with counseling centers. If necessary, external specialists (special education teacher, speech therapist, psychologist, social worker, etc.) were also involved.

#### 3.5.1. Inclusion and exclusion on the state level

At state level, we also detected efforts to increase inclusion; at the same time, we also found evidence of exclusion. At state level, increasing inclusion is mostly related to attitudes in society, which are reflected in policies and legislation. The results of this case study indicated insufficient awareness of inclusive education and its positive effects. However, this may lead to negative attitudes toward inclusion and encourage the exclusion of certain groups. At the same time, it appeared that the policies and laws of inclusion were not sufficiently clear and unambiguously documented or were too rigid to implement inclusion. Considering the specific attributes of all children (one of the principles of learning and education in the national curriculum) is one example of increasing inclusion. Counseling centers had also been set up at the local government level, and the support center Rajaleidja was established by the state in order to help kindergartens and families support children’s development and meaningful participation in early childhood education settings.

## 4. Discussion

The purpose of this study was to investigate the relevance/applicability of the model by using a case study and explore to what extent the results reflect the aim of increasing inclusion and reducing exclusion. Using several methods in the study provided a holistic description and better insight into the implementation of inclusive education, ensuring that all key characteristics and features describing these key characteristics would be detected. Every method had its specificity in the context of the study and allowed us to collect data about different features.

All 14 key characteristics of the theoretical model at the five levels are also relevant in the case study. However, the visibility of the key characteristics varied depending on the method used. All key characteristics were revealed in both the questionnaire and the focus group interview. However, in the observation and document analysis, some key characteristics occurred only partially and some not at all. By partial occurrence, we mean that some features describing the key characteristics did not occur. This was mostly due to the nature of the method. For example, the observation did not allow us to see the key features of the family, institution, and state levels, as the observation was conducted in a group room.

More significant differences from the theoretical model were found in the features describing the key characteristics at each level in more detail. Whereas some features did not appear in this case study at all, some new features were added to the model. At the family level, the study did not allow us to see the feature *perception of inclusive education*. At the institution and state levels, *philosophy of inclusion* did not appear, which suggests that the philosophical sense of inclusive education has not received meaningful consideration and has remained unclear. The principal focus is on the practical nature of inclusive education, whereas the definition of inclusive education contains both dimensions—the philosophical and the practical sense. Florian (2014) argues that developing a universally accepted definition may represent a positive step toward developing an inclusive practice. Kivirand et al. (2022) found in their case study that the development activities planned for implementing inclusion depended to a significant degree on how the institution understood the concept of inclusive education. They also focused on how to increase the capacity of the whole institution to put inclusive education into practice. Thus, to raise public awareness, the nature of inclusive education and its positive effects should be discussed much more in society.

We also added some new features to the model based on this case study. At the teacher level, we added the feature *teacher’s professional development* as a *teacher attribute*, with teachers’ continuous professional development revealed as an essential prerequisite for the implementation of inclusive education and its quality. Some researchers (Sharma et al., 2019; Chadwell et al., 2020) also indicate teachers’ insufficient preparation as a barrier to implementing inclusive education. The study revealed a lack of teachers’ specific skills in supporting special needs children and kindergarten staff’s low awareness of inclusive education. Staff and staff-related resources are very important indicators for implementing inclusive education (Sharma et al., 2019; Locke et al., 2020). Therefore, we specified at the institution level the feature presented in the theoretical model as *personnel-related activities*. The case study indicated that more detailed features could be presented separately here: *structure of employees (positions, number of employees, additional workforce); work organization (work schedules, workload, work tasks); and benefits (benefits, bonuses, rest time, and holidays)*. These features derived from the local context, and participants perceived these features as problems regarding the implementation of inclusive education.

The case study highlights the state-level challenges of implementing inclusive education, as do several other studies (Grisham-Brown et al., 2010; Schuelka, 2018; Magnússon et al., 2019; Haug, 2020). The main concern concerns *policy* and the general understanding of inclusion (Magnússon et al., 2019; Haug, 2020). Regarding the *local policy and laws of inclusion*, it was apparent in our study that the principles of inclusive education were not sufficiently clear and unambiguously documented or were too rigid to implement inclusion. Sharma et al. (2019) indicate the lack of resources as a barrier to the implementation of inclusive education. Lack of support specialists such as special education teachers and speech therapists in the society and insufficiency of resources was evident in our study as well. Unlike other studies, however, ours detected ineffective *cooperation* in the support system outside the kindergarten. State-level *monitoring and evaluation* that did not include *stakeholders’ opinions* was also a challenge. As mentioned above, a shared understanding of inclusive education is a prerequisite for its implementation. Therefore, it is important to initiate more discussion on this issue, both in the media and with key persons in the education system. This would help consider stakeholders’ opinions, move toward more inclusive education policies, and create clearer and unambiguously documented laws to support the implementation of inclusive education in the best possible way.

The model of inclusive education consists of key characteristics on all levels of the education system, thereby requiring responsibility at different levels. Thus, it will help to implement inclusion in early childhood education. Although the main idea of inclusive education is to increase inclusion and reduce exclusion, this may not always be the case. Thus, to promote inclusion and reduce exclusion, it is necessary to pay attention to very different aspects of educational contexts (Booth and Ainscow, 2002; Slee, 2005). The results of this case study suggest that the concept of inclusive education is mainly referred to in its narrow meaning. When implementing inclusive education, the focus is mostly on special needs children. Children with, e.g., different linguistic or cultural backgrounds tend to remain somewhat unnoticed. This might be traced back to the local context since, in the past, education in Estonia used to be segregated in the sense that children with special needs were taught separately from normally developed children in special kindergartens or special groups. As long as teachers keep to the concepts of mainstream and special needs children, the concept of inclusive education cannot be implemented in its truest intent (Slee, 2005). This case study showed that policy and legislation do not currently support inclusion, as the relevant regulations are not clear and straightforward enough or are too rigid. According to Booth and Ainscow (2002) and Ballard (2013), legislation and the language used when talking about inclusive education can lead to exclusion rather than inclusion. Therefore, how we organize teaching and learning activities, how we talk about inclusive education, and how we present it in legislation are of crucial importance. Ballard (2013) suggests that to reduce exclusion and create more inclusive ways of living, we need to think differently and consider alternative ideas that would support the development of more fair and democratic practices.

## 5. Conclusion

The findings from this study have implications with regard to inclusive classrooms in early childhood education. Based on our research, we see that inclusive education, as it has been operationalized in early childhood education, does not always lead to inclusion. Therefore, further studies should focus on describing practices that help to achieve better inclusion and avoid exclusion at every level according to the inclusive education model used in our study. In addition, it might be valuable to study how different interventions could have an effect on teachers’ practices and the inclusion of children.

## 6. Limitations

There are some limitations to consider. First, the number of participants was small, and therefore, any generalizations should be made with some caution. Secondly, as the results of this case study are relevant in the context of Estonia, one should be careful when interpreting these results in an international context.

## Data availability statement

The raw data supporting the conclusions of this article will be made available by the authors, without undue reservation.

## Ethics statement

The studies involving human participants were reviewed and approved by Research Ethics Committee of the University of Tartu. Written informed consent to participate in this study was provided by the participants’ legal guardian/next of kin.

## Author contributions

PN contributed to the conception and design of the study, prepared and conducted data collection and analysis, and wrote all sections of the manuscript. MP contributed to the conception and design of the study, the final data analysis process, and the editing and reviewing of the manuscript. CŠ contributed to data analysis as the co-coder and edited the manuscript. All authors contributed to the article and approved the submitted version.

## Funding

This research was supported by the EEA and Norwegian financial instruments under grant number 36.1-3.4/289.

## Conflict of interest

The authors declare that the research was conducted in the absence of any commercial or financial relationships that could be construed as a potential conflict of interest.

## Publisher’s note

All claims expressed in this article are solely those of the authors and do not necessarily represent those of their affiliated organizations, or those of the publisher, the editors and the reviewers. Any product that may be evaluated in this article, or claim that may be made by its manufacturer, is not guaranteed or endorsed by the publisher.
